# Myths and realities in the management of the open abdomen with negative pressure systems. A case report and literature review

**DOI:** 10.1016/j.ijscr.2019.07.047

**Published:** 2019-07-22

**Authors:** J. Aguilar-Frasco, P. Moctezuma-Velázquez, J.H. Rodríguez-Quintero, F.U. Pastor-Sifuentes, E. Sanchez Garcia-Ramos, U. Clemente-Gutierrez, J. Morales-Maza, O. Santes, J.D. Hernández-Acevedo, E. Contreras-Jimenez, S. Mier y Terán

**Affiliations:** General surgery department, Instituto Nacional de Ciencias Médicas y Nutrición “Salvador Zubirán”, Ciudad de México, Mexico

**Keywords:** Open abdomen, Negative pressure therapy, Abdominal sepsis, V.A.C therapy

## Abstract

**Introduction:**

The open abdomen is a useful resource for treating patients with abdominal hypertension and abdominal compartment syndrome. Currently, early closure assisted with negative pressure devices is considered standard of treatment, and its use has demonstrated favorable outcomes and a decreased rate of complications.

**Presentation of a case:**

We present a case of a 32-year-old male patient with diagnosis of non-seminomatous germinal testicular tumor (Stage IIIB (T3-N3-M1), which was summited to surgery, as a complication he presented massive bleeding, that culminated in acute compartment syndrome. With the aforementioned findings the patient re entered the operating room and was managed with open abdomen combined with a medial retraction technique of the abdominal fascia. Currently, the patient has not presented recurrence or late complications after a year.

**Discussion:**

The use of negative pressure techniques for open abdomen management began to be generalized in 1995. Subsequently, this technique evolved to V.A.C therapy (Vacuum-assisted closure therapy). Currently, these negative pressure techniques have become the most used method for the temporary closure and management of open abdomen. Controversies continue to limit its widespread use and effectiveness.

**Conclusion:**

Adequate application of negative pressure therapy in combination with techniques of medial retraction of the abdominal fascia, have proved to be useful in management for patients with open abdomen.

## Highlights

• The open abdomen is a useful resource for treating patients with abdominal hypertension and abdominal compartment syndrome.
• Multiple techniques have been described in the literature.
• Adequate application of negative pressure therapy in combination with fascial retraction, has proved to be the most convenient approach in the management of the open abdomen.

## 1 Introduction

The first surgeon to describe the use of open abdomen (OA) was Andrew J. McCosh in 1897, as a technique for the management of patients with secondary peritonitis [1,2]. This therapeutic option was unusual at that time, after that, its use became popular in surgery for damage control and as a measure to prevent abdominal compartment syndrome (ACS). Since then, multiple conditions and clinical situations have shown favorable results when treated with an open abdomen such as intestinal edema following excessive resuscitation, shock or massive bleeding, abdominal trauma, patients with ruptured abdominal aortic aneurysm and patients with intraabdominal infections or severe pancreatitis [3,4].

**Fig. 1:**
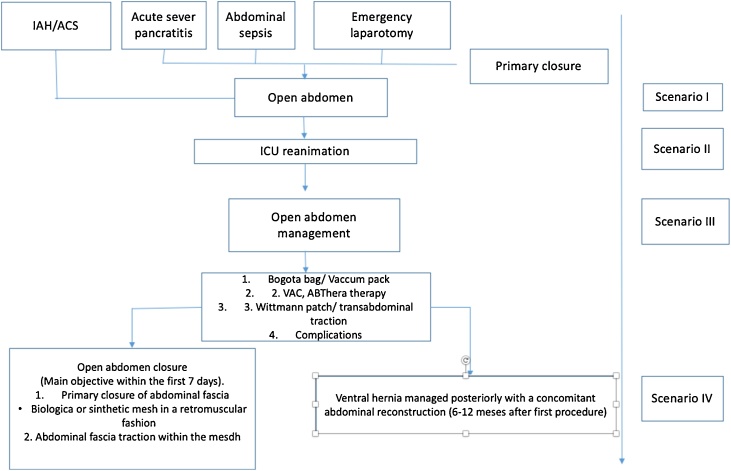
Open abdomen management diagram.

The mortality rate in patients with OA usually exceeds 30%, depending on the cohort consulted. These critically ill patients require a standardized and multidisciplinary management that includes at least surgeons and intensivist [5] (Fig. 1). This work has been reported in accordance with the SCARE criteria [6].

Different techniques for the management of OA have been described, including the Bogota bag, Wittmann patch and negative pressure systems (Vacuum-assisted closure therapy -V.A.C, ABThera System). Likewise, some authors have reported multiple combinations of these methods, currently, negative pressure systems in conjunction with maneuvers that prevent abdominal fascial retraction are considered the preferred technique in experienced centers, since they allow better control and assessment of the peritoneal fluid loss, mortality, incidence of infection and improved primary closure rates [7].

## 2 Objective

We present a clinical case of a patient with OA managed with different types of negative pressure therapy and abdominal fascial traction with synthetic mesh in a tertiary care University based center in Mexico City. In addition, we performed a narrative review about controversies in the management of open abdomen following extensive revision of literature. We included the most representative research articles in the subject finding 26 manuscripts on which we rely the recommendations described.

### 2.1 Ethical considerations

The authors declare that for this investigation there have been no experiments made in humans or animals. The study was approved by our internal Institutional Ethics Review Board, and the authors have obtained the informed consent of the patients and / or subjects referred to in the article. This document is in the possession of the correspondence author. The research committee of our Institute approved this review.

## 3 Case report

32-year-old male patient with diagnosis of non-seminomatous germinal testicular tumor (Stage IIIB (T3-N3-M1), who underwent radical orchiectomy in May 2018 followed by 7 cycles of adjuvant chemotherapy with Bleomycin, Etoposide and Cisplatin. During his follow-up a computed tomography of the abdomen and pelvis was performed, which reported the presence of multiple retroperitoneal adenopathies located towards the root of the mesentery. He was considered as a candidate for retroperitoneal lymphadenectomy. During surgery, the patient presented massive bleeding (12 liters) from a lesion of a left upper polar renal artery, and required the infusion of 11 liters of Ringer Lactate solution, norepinephrine with a maximum dose of 7 mcg / kg / min, protocol of massive transfusion (12 red blood cell packages, 9 fresh frozen plasmas and 2 platelet apheresis), use of 1 g of tranexamic acid and left nephrectomy to control acute hemorrhage.

The postoperative period was managed in the intensive care unit, where arterial hypotension, hyperlactatemia, oliguria and intra-abdominal pressure of up to 26 mmHg was documented integrating the diagnosis of ACS. With the aforementioned findings the patient reentered the operating room, with the placement of a vaccum pack.

The patient was re-intervened 72 h later for the replacement of the closure device and was also summited to the placement of fascial traction device with light polypropylene mesh.

**Fig. 2:**
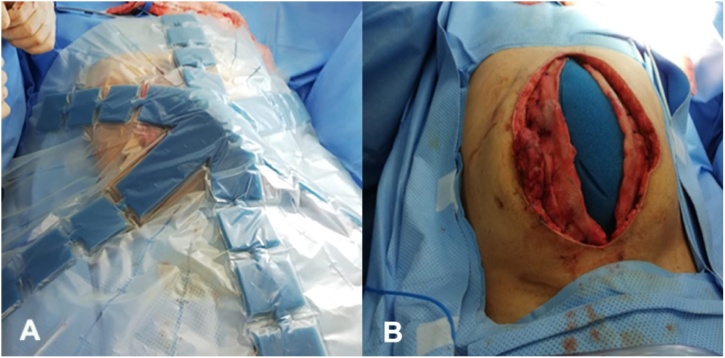
ABThera system placement. a) Protective visceral layer, b) Placement of the first retrofascial polyurethane dressing.

**Fig. 3:**
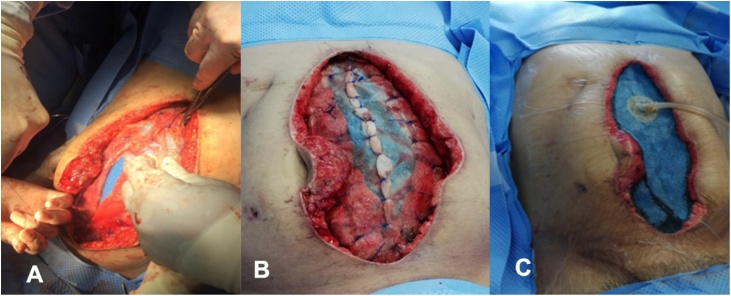
a–b) traction and closure of the mesh, c) placement of the second dressing and track to suction.

**Fig. 4:**
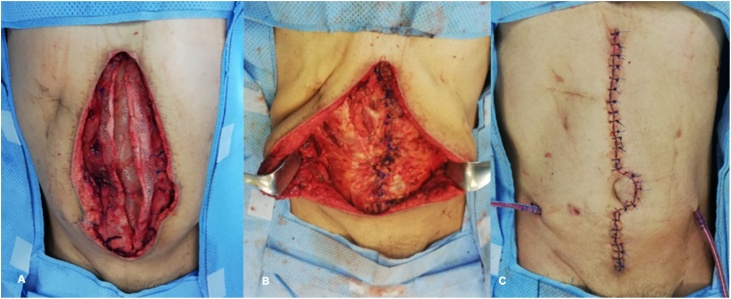
Abdominal wall closure. a–b) withdrawal of ABThera system and fascial traction mesh, c–d) anterior separation of components with a wall closure.

During the next reoperation 48 h later, the use of ABThera therapy and abdominal fascial traction began. At this moment, a 15 cm resection of ischemic distal ileum with primary mechanic anastomosis was performed (Fig. 2). On the 13th day with OA management, the ABThera system and mesh were removed and successful closure of the abdominal wall was achieved with an anterior component separation. (Fig. 3, Fig. 4). Currently, the patient has not presented any complication in one year follow up.

## 4 Discussion

The use of negative pressure techniques for OA management became popular in 1995, when Barker et al. described the use of vacuum-filled dressings for temporary closure of the abdominal cavity [8,9]. Subsequently, this technique evolved to V.A.C therapy (Vacuum-assisted closure therapy), which was described by Garner et al [10].

These negative pressure techniques have become the most used method for the temporary closure and management of OA. Although the indications and benefits of negative pressure techniques are known, controversies continue to limit its widespread use and effectiveness, in addition, we still require a standardized algorithm and solid evidence to support its usefulness. The main controversies regarding this issue are summarized below.

## 5 Negative pressure. How many millimeters of mercury are recommended?

**Fig. 5:**
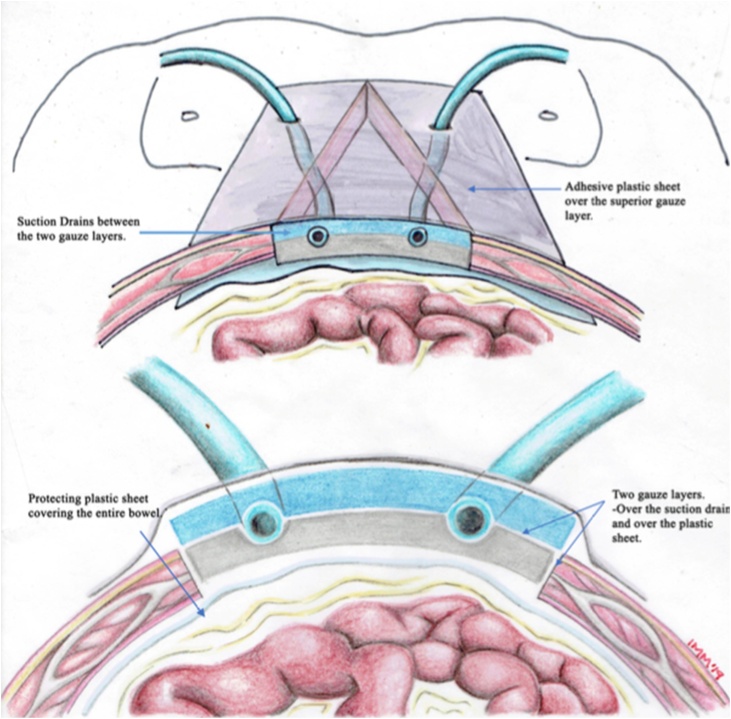
Barker technique. a) Suction drainage b) Adherent plastic cover c) Polyurethane sponge d) Polyethylene protective layer.

Current recommendations depend on the technique used, and the method to generate the negative pressure. In the technique described by Barker et al. (vacuum pack) (Fig. 5), the author recommends a negative pressure of 100–150 mmHg to maintain an optimal seal and better management of peritoneal fluid [8]. The recommended pressure for the V.A.C system (Abdominal Dressing System and ABThera) is 125 mmHg; This level of pressure has been shown to be critical for increasing blood flow, stimulating cell reproduction and maximizing the effect of negative pressure on tissue expansion [11]. Although there is no clinical evidence or randomized prospective studies that supports this information. Some authors recommend the use of lower pressures (25–50 mmHg) when there is high risk of bleeding and in the context of coagulopathy [12,13].

## 6 Type of therapy. Intermittent or continuous?

Intermittent negative pressure has been shown to increase the percentage of granulation tissue growth by 103% compared to continuous therapy (63%) in the treatment of soft tissue wounds [14]. However, the manufacturer´s recommend a continuous pressure therapy for the OA management, so it can provide constant support and tension to the abdominal fascia, facilitating its approximation. In addition, it theoretically helps to prevent the displacement of the protective layer over the intestinal loops, which can lead to intestinal erosion and the subsequent formation of fistulas. At the time of writing this article, there are no randomized clinical trials comparing intermittent versus continuous therapy for OA.

## 7 Replacement frequency of the negative pressure system

There is no sustainable evidence to support these statements there are just manufacturer's recommendations. For the Barker´s negative pressure technique (vacuum pack), the recommendation is to change it every 72–96 hours to try to minimize the exposure and manipulation of the intestinal loops [8].

In the case of the ABThera and VAC Abdominal Dressing System, the manufacturer recommends a replacement frequency of 48 to 72 h in order to prevent the granulation tissue from growing on the polyurethane dressing making it more difficult to replace it [11]. In addition, studies have shown increased growth of bacterial microorganisms when the system is replaced less frequently (>72 h), although this has not been directly related to increased rates of infectious complications.

## 8 Use of negative pressure therapy in intestinal anastomoses

It is understandable to fear the use of negative pressure therapy on a patient with OA who has undergone gastrointestinal tract resection and primary anastomosis, either manually or mechanic, due to the theoretical increase risk of anastomotic leakage or dehiscence. There are no clinical studies performed in humans that respond to this statement, however, the evidence in porcine models shows that the direct application of negative pressure (125 mmHg) on intestinal anastomosis does not increase the risk of leakage or dehiscence [15]. Therefore, performing an intestinal anastomosis is not considered a contraindication for using negative pressure systems.

## 9 Does the use of negative pressure therapy increase the incidence of enterocutaneous fistulas (EF)?

The incidence of EF in patients with OA is reported to be as high as 54.8% of cases, depending on the series [16]. One of the main concerns regarding the use of negative pressure therapy in the management of these patients is the risk of promoting the appearance of EF by applying continuous negative pressure on friable bowel. The available evidence does not support this theory [17,18]. It is well known that the most important intervention to reduce the risk of presenting EF is to close the OA as soon as possible [19].

In the case of the ABTHera dressing, the correct placement of the protective fenestrated visceral layer avoids the risk of direct contact of the intestinal loops with the polyurethane sponge. In the case of other negative pressure systems (vacuum pack and VAC Abdominal Dressing System), it is necessary to place a non-adherent layer of polyethylene (sterile plastic bag) between the intestinal loops and the negative pressure device [20].

## 10 In case of an EFs; how can an OA be managed?

Negative pressure (V.A.C therapy) has shown to be effective in the treatment of complicated wounds with the presence of EF. Specific techniques have been described in the literature that can be applied to these patients [[20], [21], [22]]. The current evidence, show that if used appropriately, VAC therapy has a double therapeutic value: 1) control of the expenditure of the EF and improvement in the wound management by controlling the enteric secretions and 2) spontaneous closure in selected cases with low morbidity and mortality [23,24].

## 11 Progressive retraction of the abdominal fascia with the use of negative pressure therapy. How to avoid it?

Although the correct application of negative pressure therapy in patients with OA has shown to reduce adhesions and intestinal loops fixation to the abdominal wall, its capacity to prevent retraction of the wound, especially the abdominal fascia, is limited. Applying continuous medial traction to the edges of the fascia prevents retraction and even allows a gradual approach. There are several commercial alternatives for this purpose (Wittman Patch, ABRA, etc.), which use Velcro material or elastic bands attached to the fascia edges achieving a success rate of closure up to 88% [25]. Similarly, polypropylene meshes have been used for this purpose, sutured to the edge of the abdominal fascia, performing progressive traction with each negative pressure therapy replacement, with acceptable results for a primary closure ranging from 68 to 76%, with low incidence of complications [25,26].

Therefore, the use of abdominal fascial traction techniques and negative pressure therapy is recommended as the technique of choice to provide the best opportunity for late abdominal primary closure in the shortest time and with minimal complications [27].

## 12 Definitive closing time of the OA

One of the dilemmas regarding to OA management is the adequate time for definitive closure, especially when the indication is abdominal sepsis. Elongated times for closure have been associated with a higher rate of complications. In general, and based on expert recommendations, abdominal closure within the first 8 days is considered to be the primary objective [22]. However, this is not easy in all cases, mainly in patients with ACS, in whom a tight closure of the abdominal fascia can increase morbidity and mortality or cause recurrence of ACS. Therefore, in this context abdominal wall closure is suggested within the first 2 weeks [[19], [20], [21]].

## 13 Conclusions

The use of OA has become a widely accepted therapeutic option in critically ill patients with severe intraabdominal pathologies. The most frequent indications for OA are abdominal trauma, peritonitis, acute pancreatitis and ACS.

In the past, the creation of a planned ventral hernia was considered an adequate clinical practice, nowadays the goal of treatment has become to achieve a late primary abdominal closure in the shortest time possible during hospitalization.

The negative pressure therapy application has gained popularity in this context, leading to a paradigm shift. Today it is used worldwide since it allows an adequate control and removal of the exudate, it prevents the accumulation of abdominal fluid and reduces the incidence of abscess formation. In addition, it decreases the progressive edema of intestinal loops, improves intestinal perfusion with few adverse effects and low morbidity and mortality.

For this reason, the adequate application of negative pressure therapy in combination with techniques of medial traction of the abdominal fascia have proved to be an adequate tool for the management of patients with OA.

## Declarations conflict of interest

The authors declare no conflict of interest.

## Funding

This research did not receive any special funding for writing or publication of this case report.

## Ethical approval

The ethical approval for the publication of this case was exempted by our institution because all of the data were collected from clinical records and imaging systems for routine perioperative planning.

## Consent

Informed consent was obtained from the patient for publication of this case report and any accompanying images.

Informed consent was obtained from the patient for publication of this case report and any accompanying images, the corresponding author has it if it is needed.

## Author contribution

All authors contributed equally.

Aguilar -Frasco, E Sanchez-Garcia Ramos, P. Moctezuma-Velázquez: Conceptualization, Methodology, Writing -original draft preparation, Investigation, Supervision. O. Santes,J. Aguilar -Frasco F.U. Pastor- Sifuentes, Emmanuel Contreras- Jimenez: Data curation, Writing- Original draft preparation. S. Mier y Terá, U. Clemente- Gutierrez, JH. Rodríguez-Quintero, Jesús Morales- Maza, J. Aguilar -Frasco Investigation, Writing- Reviewing and Editing. J. Aguilar -Frasco, J.D Hernández -Acevedo, O. Santes Visulization

## Registration of research studies

N/A.

This is not a ‘First in Man’ study and should not be registered.

## Guarantor

J. Aguilar-Frasco.

## Provenance and peer review

Not commissioned, externally peer-reviewed.

## Disclosure

Authors have no disclose
